# Investigating determinants for patient satisfaction in women receiving epidural analgesia for labour pain: a retrospective cohort study

**DOI:** 10.1186/s12871-018-0514-8

**Published:** 2018-05-09

**Authors:** Daryl Jian An Tan, Rehena Sultana, Nian Lin Reena Han, Alex Tiong Heng Sia, Ban Leong Sng

**Affiliations:** 1 Duke-NUS Medical School, 8 College Road, Singapore, 169857 Singapore; 2 Centre for Quantitative Medicine, Duke-NUS Medical School, 8 College Road, Singapore, 169857 Singapore; 3 Division of Clinical Support Services, KK Women’s and Children’s Hospital, 100 Bukit Timah Road, Singapore, 229899 Singapore; 4 Department of Women’s Anaesthesia, KK Women’s and Children’s Hospital, 100 Bukit Timah Road, Singapore, 229899 Singapore

**Keywords:** Epidural analgesia, Labour pain, Patient satisfaction, Quality measures

## Abstract

**Background:**

Epidural analgesia is a popular choice for labour pain relief. Patient satisfaction is an important patient-centric outcome because it can significantly influence both mother and child. However, there is limited evidence in the correlations between clinical determinants and patient satisfaction. We aim to investigate clinical covariates that are associated with low patient satisfaction in parturients receiving labour neuraxial analgesia.

**Methods:**

After institutional ethics approval was obtained, we conducted a retrospective cohort study using electronic and corresponding hardcopy records from 10,170 parturients receiving neuraxial analgesia between the periods of January 2012 to December 2013 in KK Women’s and Children’s Hospital in Singapore. Demographic, obstetric and anesthetic data were collected. The patient satisfaction scores on the neuraxial labour analgesia was reported by the parturient at 24 to 48 h post-delivery during the post-epidural round conducted by the resident and pain nurse. Parturients were stratified into one of three categories based on their satisfaction scores. Ordinal logistic regression models were used to identify potential covariates of patient dissatisfaction.

**Results:**

10,146 parturients were included into the study, of which 3230 (31.8%) were ‘*not satisfied*’, 3646 (35.9%) were ‘*satisfied*’, and 3270 (32.2%) were ‘*very satisfied*’. Multivariable ordinal logistic regression analysis showed that instrument-assisted vaginal delivery (*p* = 0.0007), higher post-epidural pain score (*p* = 0.0016), receiving epidural catheter resiting (*p* <  0.0001), receiving neuraxial analgesia at a more advanced cervical dilation (*p* = 0.0443), multiparity (*p* = 0.0039), and post-procedure complications headache (*p* = 0.0006), backache (p <  0.0001), urinary retention (*p* = 0.0002) and neural deficit (*p* = 0.0297) were associated with patient dissatisfaction. Chinese, compared with other ethnicities (*p* = 0.0104), were more likely to be dissatisfied.

**Conclusions:**

Our study has identified several clinical determinants that were independent associated factors for low patient satisfaction. These covariates could be useful in developing a predictive model to detect at-risk parturients and undertake time-sensitive precautionary measures for better patient satisfaction.

## Background

Epidural analgesia is a popular choice for parturients in addressing labour pains [1–4]. This provides effective pain relief with excellent safety profiles for the mother and fetus [5–7]. Given the high quality of analgesia, labour epidural analgesia could achieve high patient satisfaction [8].

Healthcare professionals have become increasingly more interested in improving long-term outcomes of the care provided. Patient satisfaction in childbirth holds important significance with respect to a patient’s outcome and experience. Having a positive experience during childbirth improves the self-esteem of mothers and their confidence in taking care of their newborns [9–11], while a negative experience promotes the risks of developing postnatal depression, poor breastfeeding and, arguably, child neglect and abuse [10–13]. Therefore, it is important to identify potentially modifiable factors which are associated with patient satisfaction so that medical professionals are better prepared to promote a better outcome. Furthermore, patient satisfaction is an indicator of the quality of healthcare; it is imperative for medical professionals to be patient-centric and to tailor therapies to achieve the optimal patient care [14–16]. Achieving high patient satisfaction in labour epidural analgesia is therefore, a reflection of the quality of epidural service delivery. However, there is a paucity of information in the literature that explores the factors influencing patient satisfaction. Hence, the aim of this study is to investigate and identify factors which are associated with low patient satisfaction in parturients receiving labour neuraxial analgesia for labour pain.

## Methods

We conducted a retrospective analysis of the labour neuraxial analgesia electronic database obtained from a single-centre at KK Women’s and Children’s Hospital, Singapore, between 01 January 2012 and 31 December 2013. The SingHealth Centralised Institutional Review Board approval was obtained prior to the commencement of the study (SingHealth CIRB Ref: 2012/259/D) and the need for informed consent was waived by the CIRB. The electronic records comprising labour neuraxial analgesia forms of 10,170 parturients were retrieved. The electronic database was thoroughly screened for missing and irregular data, and the hardcopies of the corresponding records were reviewed in this event to complete the entries. All the paturients who underwent neuraxial labour analgesia during the study period were included. According to our institution guidelines, all parturients would receive combined-spinal epidural (CSE) analgesia. However, if CSE posed much difficulty to perform, the attending anesthetist might choose to induce epidural analgesia conventionally, without the spinal analgesia component, as the next line of action. Regardless of the induction of analgesia by CSE or conventional epidural technique, analgesia was maintained through an epidural catheter with a regimen consisting of 0.1 – 0.125% of ropivacaine plus 2 μg/ml of fentanyl with a continuous infusion at a basal rate of up to 10 ml/h.

The demographic data (maternal age, body mass index and ethnicity), obstetric data (parity, pre-epidural cervical dilation and mode of delivery) and anesthetic data (type of neuraxial analgesic technique received, pre- and post-epidural pain scores, occurrence of epidural catheter resiting, total time taken to perform procedure and occurrence of breakthrough pain experienced) were collected. Breakthrough pain was defined as maternal complaints of pain or pressure that required one or more doses of unscheduled supplemental epidural medications [11]. Breakthrough pain was treated by the attending anaesthetist or resident using a typical regimen of 0.2% ropivacaine 5–10 mL, with or without fentanyl 50 mcg. Pre- and post- epidural pain scores were determined using the Visual Analogue Scale. In addition, the occurrence of complications associated with neuraxial analgesia (headache, backache, nausea/vomiting, shivering, itching, urinary retention and neural deficit) was also recorded by a standard postpartum questionnaire. Neural deficit was defined as any parturient complaint of weakness and/or numbness in the lower limbs between 24 to 48 h post-delivery and urinary retention was defined as the need for presence of indwelling urinary catheter inserted during labour and delivery still present 24 to 48 h post-delivery.

Patient satisfaction post-delivery on the neuraxial labour analgesia was defined as a numerical rating scale as a percentage (0 to 100%) reported by the parturient at 24 to 48 h post-delivery during the post-epidural round conducted by the resident and pain nurse. Patient satisfaction was routinely asked by the anesthetic service and the parturient was encouraged to provide a score. Parturients with reported satisfaction lower than 80%, between 80 and 90%, and greater than 90% were considered as ‘*not satisfied’*, *‘satisfied’* and *‘very satisfied’* respectively. These cut-offs were used as audit standards by the institution. The primary outcome of patient satisfaction was treated as ordinal categorical data.

### Statistical analysis

All demographic, clinical and anesthetic data were summarized with respect to status of patient satisfaction. Continuous variables were summarized either as mean with standard deviation (SD) or median with interquartile range (IQR) and range, and categorical variables were summarized as frequency with proportion. Univariate and multivariable ordinal logistic regression models with cumulative link models were used to identify the associations between potential covariates and level of patient’s satisfaction. The category of interest in our analysis was parturients who were *‘not satisfied’*. Associations from the ordinal logistic regression models were characterized using odds ratio (OR) and corresponding 95% confidence interval (95% CI). Variables with *p*-value < 0.2 in the univariate model were selected for the multivariable model. Then union of the variables from forward, backward and stepwise method were used to finalize the variable lists in the multivariable model with entry and stay criteria as 0.2 and 0.05 respectively. Then we used likelihood ratio test followed by area under the curve (AUC) to decide the final multivariable model. Then we explored multicollinearity using Pearson’s product moment correlation and managed by stepwise variable reduction and quantified by variance inflation factor (VIF). We fit stepwise regression method with clinically meaningful interaction effects with entry and stay criteria as 0.05 and 0.05 respectively. We also compared model with and without interaction effects for performance checking based on Akaike’s information criterion (AIC) and AUC from receiver’s operating characteristics (ROC) curve. If models with and without interaction effects has similar level of performance then based on principle of parsimony, the model without interaction effects would be selected as the final multivariable model. Significance level was set at 0.05 and all tests were two-tailed. SAS version 9.3 software (SAS Institute; Cary, NC, USA) was used for the analysis.

We had 10,171 eligible patients. Among them 1 patient had incomplete data. Out of these 10,170 patients, 10,146 patients had non-missing information of patient’s satisfaction. Our primary objective was to find associated risk factors for “very satisfied” and “satisfied” groups. Peduzzi et al., Concato et al. and Vittinghoff et al. recommended that multivariable logistic regression models should be used with at least 10 events per predictor variable [17–19]. We had 20 clinically meaningful variables to account for in the multivariate model and hence we needed at least 2*10*20 = 400 events in the data. In our data, prevalence of “satisfied” and “very satisfied” group were at least 30% in each group i.e. we had more than 3000 patients in each “satisfied” and “very satisfied” groups. Our study was adequately powered (> 90%) with 10, 146 patients based on following assumptions: proportion of “satisfied” / “very satisfied” as 30%, OR of 1.5 (or 0.67) and alpha or type I error rate as 5%.

## Results

**Fig. 1:**
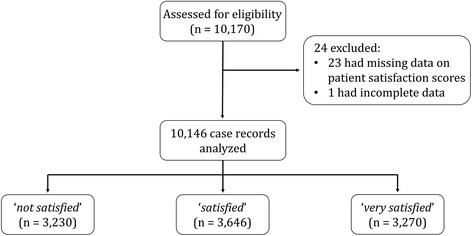
Flow chart of study

A total of 10,170 parturients were selected for the study. 23 parturients had missing data on patient satisfaction scores and one parturient had data that could not be traced, hence these parturients were not analysed. Among these 10,146 parturients, 3230 (31.8%), 3646 (35.9%) and 3270 (32.2%) were ‘*not satisfied*’, ‘*satisfied*’ and ‘*very satisfied*’ respectively. The flow chart of the study is illustrated in Fig. 1. Table 1 shows the demographics of the parturients.

**Table 1:** Characteristics of 10,146 parturients categorized based on patient satisfaction scores

	Not satisfied (*n* = 3230)	Satisfied (*n* = 3646)	Very satisfied (*n* = 3270)	Total (*n* = 10,146)
Age (years), mean (SD)	29.8 (5.11)	29.9 (4.98)	30.1 (5.0)	29.9 (5.0)
Ethnicity, n(%)				
Chinese	1573 (48.7)	1767 (48.5)	1481 (45.3)	4821 (47.5)
Indian	387 (12.0)	438 (12.0)	450 (13.8)	1275 (12.6)
Malay	760 (23.5)	861 (23.6)	756 (23.1)	2377 (23.4)
Others	510 (15.8)	580 (15.9)	583 (17.8)	1673 (16.5)
Body mass index (kg.m^−2^), mean (SD)	27.4 (5.5)	27.6 (8.4)	27.6 (4.6)	27.5 (6.5)
Parity, n(%)				
Nulliparous	1896 (58.7)	2187 (60.0)	2077 (63.5)	6160 (60.7)
Multiparous	1335 (41.3)	1457 (40.0)	1189 (36.4)	3981 (39.2)
Technique of neuraxial analgesia, n(%)				
Combined spinal-epidural	3053 (94.5)	3446 (94.5)	3110 (95.1)	9609 (94.7)
Epidural	177 (5.5)	200 (5.5)	160 (4.9)	537 (5.3)
Pre-epidural cervix dilation (cm), mean (SD)	3.5 (1.2)	3.5 (1.12)	3.4 (1.16)	3.5 (1.2)
Occurrence of epidural catheter resiting, n(%)	40 (1.2)	18 (0.5)	14 (0.3)	72 (0.7)
Total time taken to perform procedure (minutes), mean (SD)	7.3 (5.11)	6.9 (4.8)	6.7 (4.4)	7.0 (4.8)
Mode of delivery, n(%)				
Normal vaginal delivery	2371 (73.5)	2843 (78.2)	2172 (66.7)	07,386 (73.0)
Instrument-assisted vaginal delivery	0370 (11.5)	0391 (10.8)	0268 (8.2)	01,029 (10.2)
Lower-segment caesarean section	0483 (15.0)	0403 (11.1)	0816 (25.1)	01,702 (16.8)
Pre-epidural pain score, median (IQR [Min - Max])	7.0 (5 – 9 [0 – 10])	8.0 (5 – 9 [0 – 10])	7.5 (5 – 9 [0 – 10])	7.0 (5 – 9 [0 – 10])
Post-epidural pain score, median (IQR [Min - Max])	0 (0 – 0 [0 – 9])	0 (0 – 0 [0 – 9])	0 (0 – 0 [0 – 8])	0 (0 – 0 [0 – 9])
Reported post-epidural complications, n(%)				
Headache	26 (0.8)	27 (0.7)	4 (0.1)	57 (0.6)
Backache	328 (10.2)	283 (7.8)	123 (3.8)	734 (7.2)
Nausea/Vomiting	318 (9.8)	359 (9.8)	331 (10.1)	1008 (9.9)
Shivering	853 (26.4)	900 (24.7)	804 (24.6)	2557 (25.2)
Itching	1175 (36.4)	1269 (34.8)	1155 (35.3)	3599 (35.5)
Urinary retention	97 (3.0)	79 (2.2)	52 (1.6)	228 (2.2)
Neural deficit	14 (0.4)	11 (0.3)	4 (0.1)	29 (0.3)
Occurrence of breakthrough pain, n(%)	462 (14.3)	517 (14.2)	451 (13.8)	1430 (14.1)

Univariate and multivariable ordinal logistic regression models are presented in Table 2 to reflect the associations between potential covariates and patient satisfaction with reference to *‘very satisfied’* category. Univariate ordinal logistic regression model showed that age, ethnicity, mode of child delivery, multiparity, longer time taken to complete neuraxial analgesia, occurrence of epidural catheter resiting, higher post-epidural pain score, and occurrence of post-epidural complications of headache, backache, neural deficit and urinary retention were associated factors for lower patient satisfaction (‘*not satisfied’*, *‘satisfied’* categories).

**Table 2:** Univariate and multivariable ordinal logistic regression analyses of covariates of patient dissatisfaction. Status ‘*very satisfied*’ was used as the reference category

Characteristics	Univariate analysis			Multivariable analysis		
	Beta coefficient (SE)	OR (95% CI)	*P* – value	Beta coefficient (SE)	OR (95% CI)	*P* – value
Age (years)	−0.01 (0.003)	0.992 (0.985 – 0.999)	0.0215			
Ethnicity (Ref: Chinese)			0.0060+			0.0104+
Indian	−0.16 (0.06)	0.853 (0.761 – 0.956)	0.0064	−0.16 (0.06)	0.853 (0.760 – 0.957)	0.0066
Malay	−0.04 (0.05)	0.961 (0.878 – 1.052)	0.3875	−0.04 (0.05)	0.959 (0.876 – 1.051)	0.3739
Others	−0.14 (0.05)	0.865 (0.781 – 0.959)	0.0057	−0.13 (0.05)	0.876 (0.790 – 0.971)	0.0118
Mode of delivery (Ref: NVD)			< 0.0001+			< 0.0001+
Instrument-assisted	0.16 (0.06)	1.179 (1.046 – 1.328)	< 0.0001	0.21 (0.06)	1.237 (1.095 – 1.398)	0.0007
LSCS	−0.56 (0.05)	0.573 (0.518 – 0.634)	< 0.0001	−0.47 (0.05)	0.628 (0.566 – 0.697)	< 0.0001
Multiparity (Ref: No)	0.15 (0.04)	1.166 (1.083 – 1.254)	< 0.0001	0.11 (0.04)	1.119 (1.037 – 1.208)	0.0039
Epidural analgesia (Ref: CSE)	0.09 (0.08)	1.089 (0.929 – 1.277)	0.2913			
Total time taken to perform procedure (minutes)	0.02 (0.004)	1.019 (1.012 – 1.027)	< 0.0001			
Pre-epidural cervix dilation (cm)	0.06 (0.02)	1.06 (1.027 - 1.094)	0.0003	0.03 (0.02)	1.034 (1.001 – 1.067)	0.0443
Occurrence of epidural catheter resiting (Ref: No)	0.92 (0.23)	2.499 (1.587 – 3.937)	< 0.0001	1.02 (0.23)	2.775 (1.752 – 4.396)	< 0.0001
Pre-epidural pain score	−0.002 (0.006)	0.998 (0.987 - 1.009)	0.7400			
Post-epidural pain score	0.09 (0.03)	1.094 (1.024 – 1.151)	0.0058	0.08 (0.03)	1.085 (1.031 – 1.142)	0.0016
Occurrence of breakthrough pain (Ref: No)	0.03 (0.05)	1.032 (0.931 - 1.143)	0.5532			
Reported post-epidural complications (Ref: No)						
Headache	0.83 (0.24)	2.295 (1.437 – 3.664)	0.0005	0.83 (0.24)	2.296 (1.432 – 3.679)	0.0006
Backache	0.69 (0.07)	1.995 (1.737 – 2.291)	< 0.0001	0.61 (0.07)	1.840 (1.599 – 2.117)	< 0.0001
Nausea/Vomiting	- 0.02 (0.06)	0.977 (0.867 – 1.102)	0.7076			
Shivering	0.07 (0.04)	1.074 (0.989 – 1.166)	0.0912			
Itching	0.03 (0.04)	1.034 (0.960 – 1.115)	0.3759			
Urinary retention	0.48 (0.12)	1.611 (1.263 – 2.056)	0.0001	0.46 (0.13)	1.591 (1.242 – 2.039)	0.0002
Neural deficit	0.79 (0.34)	2.205 (1.122 – 4.333)	0.0218	0.76 (0.35)	2.134 (1.077 – 4.228)	0.0297

*NVD* normal vaginal delivery, *LSCS* lower-segment caesarean section, *CSE* combined spinal-epidural

Multivariable model with interaction and without interaction had similar AUC and AIC values, hence we chose model with main effects only. Multivariable ordinal logistic regression model showed that parturients who were Chinese (*p* = 0.0104) and multiparous (*p* = 0.0039) were more likely to report post-delivery dissatisfaction. Covariates in the intrapartum period that were associated with lower patient satisfaction were parturients who received instrument-assisted vaginal delivery (*p* = 0.0007), resiting of epidural catheter (*p* <  0.0001), neuraxial analgesia at a more advanced cervical dilation (*p* = 0.0443), and parturients who experienced higher post-epidural pain score (*p* = 0.0016). Furthermore, occurrences of post-epidural complications of headache (*p* = 0.0006), backache (*p* <  0.0001), urinary retention (*p* = 0.0002) and neural deficit (*p* = 0.0297) were associated with low patient satisfaction. Interestingly, parturients who received lower-segment caesarean section (LSCS) for child delivery were more likely to report higher patient satisfaction (*p* <  0.0001).

## Discussion

We found that experiencing higher post-epidural pain score, having instrument-assisted vaginal delivery, receiving epidural catheter resiting, and receiving neuraxial analgesia at a more advanced cervical dilation were intrapartum factors associated with low patient satisfaction. Post-procedure complications such as headache, backache, urinary retention and neural deficit were associated with low patient satisfaction. Chinese ethnicity and multiparous parturients were also more likely to report low patient satisfaction.

The knowledge of these clinical associated factors for patient dissatisfaction could be useful to the attending anesthetist to risk-stratify parturients at higher risk of reporting low patient satisfaction and institute strategies to improve clinical outcomes. The importance of patient satisfaction in labour neuraxial analgesia could reflect the quality of analgesia, including the effectiveness and side effects related to neuraxial analgesia; this could have a bearing on the likelihood of the mother to be more receptive to regional anesthesia in future deliveries. Dharmalingam et al. showed, in a cross-sectional study involving 200 women who have received spinal anesthesia for caesarean section, that 194 (97%) parturients were satisfied with the spinal anesthesia and 177 (88.5%) of them would opt for spinal anesthesia in the future for similar surgery if required [20]. This finding also corroborated with other studies [21, 22]. On the other hand, Charuluxananan et al. showed that patient refusal of neuraxial anesthesia was correlated with a low patient satisfaction score [23]. These demonstrate the relationship between patient satisfaction and the likelihood to receive neuraxial anesthesia in future. However, although analgesic effectiveness contributes to the satisfaction with labor analgesia care, it is not the only contributor to patient satisfaction [24].

Ethnic difference in patient satisfaction was found in our study. Chinese was used as the reference ethnic group because it was the majority of the study population. When compared against Malays, Chinese parturients were less likely to report higher patient satisfaction. Several studies have suggested interethnic differences on the outcomes of epidural analgesia in parturients [25–27]. Cultural differences could lead to women perceiving the process of childbirth, coping mechanisms and labour pains differently, thereby leading to the differences in patient satisfaction [27, 28]. In the non-obstetric setting, it has been shown that pain perception was underestimated in ethnically diverse populations [29, 30]; our study implies a similar picture might be occurring in the obstetric setting in terms of patient satisfaction.

Pain experienced whilst being on labour neuraxial analgesia is a significant association factor for low patient satisfaction. Furthermore, Carvalho et al. found that pain experienced during and after caesarean delivery were important patient concerns [31]. Not surprisingly, our results showed that higher post-epidural pain scores experienced by the parturient were associated with lower patient satisfaction. A likely explanation to this could be parturients’ expectations of minimal to no pain experienced after receiving neuraxial analgesia, which when they fall short of, result in lower patient satisfaction.

We found that multiparous parturients, when compared with nulliparous parturients, were more likely to be dissatisfied after receiving neuraxial analgesia. This association has not been well studied, and there are differing views with regards to how parity affects patient satisfaction. Koteles et al. has shown in their study that multiparous women were less receptive towards epidural analgesia and were less likely to use it [32]. However, Bélanger-Lévesque et al. has shown in their cross-sectional study of 200 mothers that parity could suggest higher patient satisfaction, as prior experience increased their preparation for their current childbirth [33].

Parturients who had instrument-assisted vaginal deliveries were more likely to be dissatisfied as compared to parturients who had normal vaginal deliveries. Our findings were corroborated by a cluster analysis study performed by Rudman et al. that reported negative correlations between patient satisfaction and incidence of instrument-assisted vaginal delivery [34]. It is likely that instrument-assisted vaginal delivery is an indicator of difficult labour, and parturients could have experienced more pain.

Interestingly, we found that parturients with increased cervical dilation at the onset of neuraxial analgesia administration were more likely to be dissatisfied. The initiation of neuraxial analgesia in early labour has been found to be associated with an increased maternal satisfaction and shortened first stage of labour, without increasing the risk of caesarean deliveries [35]. The provision of neuraxial analgesia later in labour could also be associated with lower pain control for a longer period of time prior to receiving neuraxial analgesia.

The occurrence of epidural catheter resiting reported in our study was 0.71%, lower than those reported in previous studies [36, 37]. We found that patients who underwent epidural catheter resiting were more likely to be dissatisfied. As with any invasive procedure, epidural catheter resiting poses considerable distress to parturients and could in itself be perceived as an adverse event in labour epidural analgesia. The repeated procedure also introduces a greater risk of complications in addition to a higher financial cost. Furthermore, the resiting of the epidural catheter is usually performed when the catheter malfunctions or is misaligned. As such, the earlier epidural catheter could have provided inadequate analgesia and resulted in poor pain relief and patient distress, prior to the resiting procedure. Hence, catheter resiting could be a surrogate marker for inadequate analgesia that contributed to a lower patient satisfaction.

The presence of complications after receiving neuraxial analgesia was significantly associated factors relating to low patient satisfaction [22, 38]. It is important to be cognizant of these postoperative complications not only because they could lead to lower patient satisfaction in the current pregnancy, but also increase the tendency of refusing labour neuraxial analgesia in future pregnancies [39]. Of note, the association of post-delivery backache with neuraxial block has not been conclusively determined even though it may be perceived as otherwise [40–42]. Hence, education on post-delivery backache should be included in maternal informed consent as it may potentially help manage parturients’ expectations and remove misconceptions.

Nevertheless, we acknowledge the limitations that exist in our study. Patient satisfaction is a multidimensional parameter which could be related to patient, obstetric, anesthetic and psychological factors. Parturients could have reported higher patient satisfaction scores when asked by the anesthetic service as they might have felt obliged to give a higher patient satisfaction score while they were still hospitalized and might potentially require further services from the attending anesthetist [20, 38]. We tried to mitigate this risk by having patient satisfaction scores obtained in this study 24 to 48 h after delivery by a different anesthetist who had not been directly involved in the anesthetic care of the parturient. Furthermore, recall bias could be a significant factor. Furthermore, satisfaction is multidimensional and could be influenced by many factors such as family support, environmental changes and mood changes [43], many of which were not accounted for in this study despite the large study population that conferred a good statistical power.

## Conclusion

In conclusion, we found several clinical determinants in the provision of neuraxial analgesia that may have significant influences on patient satisfaction in this study. These determinants are important for the anesthetist to risk-stratify parturients, with further data analytics, as there are time-sensitive opportunities for necessary measures to enhance better patient satisfaction and the quality of holistic care. Further studies are needed to determine if modification of these risk factors affects patient satisfaction scores.

## Abbreviations

CI: Confidence interval
CIRB: Centralised Institutional Review Board
CSE: Combined-spinal epidural
IQR: Interquartile range
LSCS: Lower-segment caesarean section
NVD: Normal vaginal delivery
OR: Odds ratio
SD: Standard deviation
